# Structures and electronic properties of domain walls in BiFeO_3_ thin films

**DOI:** 10.1093/nsr/nwz101

**Published:** 2019-07-10

**Authors:** Huaixun Huyan, Linze Li, Christopher Addiego, Wenpei Gao, Xiaoqing Pan

**Affiliations:** 1 Department of Materials Science and Engineering, University of California, Irvine, CA 92697, USA; 2 Department of Physics and Astronomy, University of California, Irvine, CA 92697, USA; 3 Irvine Materials Research Institute, University of California, Irvine, CA 92697, USA

**Keywords:** domain wall, BiFeO_3_, ferroelectric, conductivity, photovoltaics, thin film

## Abstract

Domain walls (DWs) in ferroelectrics are atomically sharp and can be created, erased, and reconfigured within the same physical volume of ferroelectric matrix by external electric fields. They possess a myriad of novel properties and functionalities that are absent in the bulk of the domains, and thus could become an essential element in next-generation nanodevices based on ferroelectrics. The knowledge about the structure and properties of ferroelectric DWs not only advances the fundamental understanding of ferroelectrics, but also provides guidance for the design of ferroelectric-based devices. In this article, we provide a review of structures and properties of DWs in one of the most widely studied ferroelectric systems, BiFeO_3_ thin films. We correlate their conductivity and photovoltaic properties to the atomic-scale structure and dynamic behaviors of DWs.

## INTRODUCTION

Since the discovery of ferroelectricity in BaTiO_3_ in the mid 1940s [1], ferroelectric oxides have become a prototypical example of functional materials, attracting considerable interest both in fundamental research and device engineering. The spontaneous polarization in a ferroelectric can be reversed through the application of an external electric field that is greater than the coercive field, a behavior similar to the reorientation of magnetic moments under an applied magnetic field for ferromagnetic materials. Due to such switchability and the small size of ferroelectric domains, which can be as small as a few nanometers [2], ferroelectric materials can be utilized in an important class of high-density and non-volatile memories. Ferroelectric memories have a number of advantages, such as low power consumption, fast writing speed, and high cyclability, which, in most cases, is superior to the performance of other non-volatile devices [3].

The domain wall (DW) is an important element of ferroelectric materials. It is a quasi-2D boundary separating domains that differ in the orientation of spontaneous polarizations. The DW types are distinguished by the rotation angles of polarization between neighboring domains, e.g. 71°, 109°, and 180° in BiFeO_3_. DWs appear as the material is cooled below the Curie temperature (*T*_C_) [4]. This occurs as a result of symmetry breaking in the crystal structure during the phase transition, and the domain pattern that forms is governed by minimization of the free energy of the boundaries [5,6]. This free energy is related to electrostatic energy and elastic energy, which can be affected by several factors. Polarization bound charges and free charges [7–10] affect electrostatic energy, misfit strain contributes to elastic energy [10], and defects [11–14] affect both electrostatic and elastic energy. Variation of these factors can lead to the stabilization of a number of unique DW structures, either ordered or disordered. Over the past few years, there has been continuously increasing evidence showing that DWs can exhibit novel properties, including enhanced conductivity [15–19], photovoltages [20–26] and current rectification [27], all of which make DWs appealing for applications as active elements in future nanodevices.

In this review, we summarize the recent progress on the experimental study of DW structures and properties in one of the most widely studied ferroelectric systems, BiFeO_3_. We will first illustrate the microstructure of 71°, 109° and 180° DWs in BiFeO_3_ thin films, and the engineering methods used to produce ordered DW patterns, including boundary-condition engineering and defect engineering. The microstructures and atomic structures of DWs are revealed by a combination of piezoresponse force microscopy (PFM), aberration-corrected atomic-resolution scanning transmission electron microscopy (STEM), and *in situ* transmission electron microscopy (TEM). This is followed by a discussion on the properties of the DWs including DW conductivity and DW photovoltaics. The studies on DW conductivity include those findings from uncharged and charged DWs, and the formation of charged domain walls (CDWs) controlled by an external electric field. We will then show that characterization of the microstructure can provide us with a fundamental understanding of the properties at the macro scale and associated structural mechanisms. Finally, we will discuss the new possibilities brought by the recent advancement of electron microscopy techniques including high-energy-resolution electron energy loss spectroscopy (EELS) and multi-dimensional imaging, on the study of the electronic structure, charge and their interplay at the ferroelectric DWs and ferroelectric materials in general.

## DW STRUCTURES AND BOUNDARY-CONDITION ENGINEERING OF DW PATTERNS

**Figure 1.**
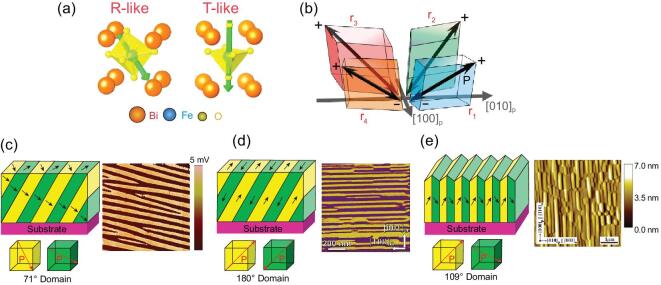
a) Atomic models of R-like and T-like unit cell structures in BiFeO_3_. Reprinted with permission from Li *et al*. [31]. Copyright (2017) American Chemical Society. b) Four variants of the BiFeO_3_ pseudocubic unit cell. Reprinted with permission from Nelson *et al*. [32]. Copyright (2011) American Chemical Society. c), d) and e) are schematics of different DWs with corresponding in-plane PFM phase mappings: periodic domain patterns of c) 71°, reprinted with permission from Bhatnagar *et al*. [22]. Copyright (2013) Springer Nature; d) 180°, reprinted with permission from Chen *et al*. [42]. Copyright (2015) American Chemical Society; e) 109°, reprinted with permission from Folkman *et al*. [54]. Copyright (2009) AIP Publishing.

BiFeO_3_ bulk material has a rhombohedral structure consisting of two pseudocubic (P) perovskite unit cells connected along the body diagonal with the two oxygen octahedra rotated clockwise and counterclockwise around the axis by 13.8° [28]. BiFeO_3_ thin films with misfit strain less than 4.5% [29] exhibit a monoclinically distorted structure that is similar to the bulk rhombohedral phase, and are thus referred to as ‘*rhombohedral*-like (R-like)’ structures [30], as shown in Fig. 1a [31]. In pseudocubic unit cells of the R-like structure, the oxygen octahedra and the central Fe cation are displaced from their respective positions at the face and body centers, giving rise to a large spontaneous polarization (∼100 μC cm^−2^) along the < 111>_P_ (subscripts P denote pseudocubic indices) directions, and resulting in four different ferroelastic variants (r_1_–r_4_) (Fig. 1b) [32]. Rotations between polarization variants in the R-like BiFeO_3_ can be 71° (ferroelastic–ferroelectric), 109° (ferroelastic–ferroelectric), or 180° (ferroelectric), yielding three types of domain walls. When the misfit strain is larger than 4.5%, the BiFeO_3_ unit cell will transform into a ‘*tetragonal*-like (T-like)’ structure [30], as shown in Fig. 1a. In such a structure, the Fe atom is coordinated by five oxygen atoms and displaced from the body center, resulting in a giant polarization of ∼ 150 μC/cm^2^ [33].

Thanks to the recent advances in atomic-level control of thin-film growth techniques, high-quality ferroelectric thin-film epitaxial growth can now be achieved with precise control over composition, interfaces, and defect introduction [34–39]. The ability to synthesize high-quality films has enabled a steady growth in the atomic-level study of the physical [31,40,41] and electrical properties [14–21,42] of ferroelectric materials.

In ferroelectric and ferroelastic thin films, patterns of DWs depend strongly on the boundary conditions at the surfaces or interfaces. First, the lattice mismatch between the film and substrate leads to a biaxial-strain mechanical boundary condition [37,43–45], which can be altered by choosing substrates spanning a wide range of lattice parameters [29,32,46–48]. Second, the electrical boundary condition is critically dependent on free charge compensation at the interfaces and can be tailored by choosing substrates or epitaxial buffer layers with different conductivities [49–51]. Additional restrictions on the boundary condition can be made by changing the vicinality or atomic termination of the substrates [52,53]. By modifying these boundary conditions, previous experimental studies demonstrated the capability to fabricate different types of ordered DW patterns (i.e. 109°, 71°, and 180° DWs) in BiFeO_3_ thin films (Fig. 1c, d and e) [22,42,54].

### 71° and 109° domain walls

Synthesis of epitaxial BiFeO_3_ thin films containing periodically ordered patterns of 71° and 109° DWs on a SrRuO_3_ layer on orthorhombic DyScO_3_ substrate was reported by Chu *et al*. in 2006 [5]. By careful control of the growth for the SrRuO_3_ layer, the in-plane lattice parameters of SrRuO_3_ films are pinned by the DyScO_3_ substrate. Consequently, the in-plane lattice parameters of BiFeO_3_ films are also pinned by the DyScO_3_ substrate and such constraints imposed by the orthorhombic substrate result in the stabilization of periodic twinning domain patterns in the BiFeO_3_ films. The observed domain patterns develop along either the [100]_P_ or [101]_P_ direction [4], which correspond to 109° or 71° DWs, respectively. In Chu's follow-up works [52,55], it was demonstrated that the type of DWs in the as-grown 100-nm-thick BiFeO_3_ films can be controlled by varying the thickness of an SrRuO_3_ conducting buffer layer between the ferroelectric and the substrate. PFM measurements show that the 109° DWs are dominant when the SrRuO_3_ thickness is less than 5 nm; DWs are a mixture of 109° and 71° when the SrRuO_3_ thickness is between 5 and 25 nm; and only 71° DWs exist when the SrRuO_3_ thickness is above 25 nm. The underlying mechanism can be attributed to the interaction of the polarization bound charge at the interface and free charge carriers in the SrRuO_3_ electrode. In particular, the 71° domain structures have a single out-of-plane polarization vector resulting in uniform positive and negative bound charge on opposite surfaces of the film. This bound charge can produce a large depolarization field, which would destabilize the domain structure unless it can be screened by free charge carriers. It follows that the 71° domain structures are usually stabilized in BiFeO_3_ thin films with the insertion of thick (>25 nm) bottom electrodes [55–57]. In contrast, if the surfaces of BiFeO_3_ thin films are uncompensated by free charges, the system favors the formation of 109° DW° patterns with alternating out-of-plane polarization that can reduce the total electrostatic energy.

### 180° domain walls

The observation of periodically ordered 180° DWs in BiFeO_3_ thin film was reported by Chen *et al*. in 2015 [42], where they studied 32-nm-thick epitaxial (110)_P_ BiFeO_3_ thin films grown on orthorhombic GdScO_3_ (010)_o_ (subscripts o denote orthorhombic indices) substrate. Based on PFM measurements, the structures are composed of periodic stripe domains separated by 180° DWs that locate at (11$\bar{2}$) and intersect with the film surface along the [100]_P_ direction. Their results also show that, as the film thickness increases to 70 nm, 71° DWs become dominant. The domain pattern formation strongly depends on the elastic and electrostatic boundary conditions. The depolarization field, which corresponds to electrostatic energy, is inversely proportional to the film thickness and can be compensated by free charges from conducting electrodes. In Chen's work, the BiFeO_3_ was directly grown on insulating GdScO_3_ substrate with insufficient free charges to fully compensate the depolarization field. In thinner films, like the 32-nm-thick film, 180° domain patterns with alternating out-of-plane polarization are formed to reduce the electrostatic energy dominated by the depolarization field. The 180° domains, however, are non-ferroelastic and could not release the strain imposed by the substrate. When the film thickness increases to 70 nm, the depolarization field is reduced; meanwhile the elastic effects start to dominate, resulting in the formation of 71° ferroelastic domains to reduce the elastic energy of the system [52,58,59].

### Vortex domains

**Figure 2.**
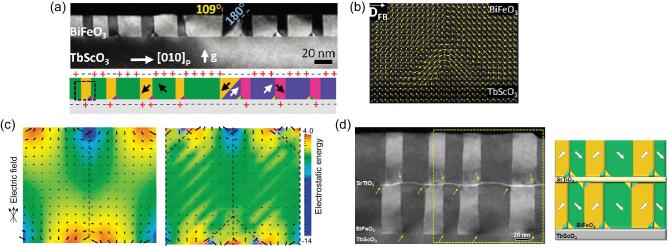
a) Dark-field TEM and corresponding schematic. b) Polarization mapping of 109° DWs that form a vortex domain. c) Vector plots of depolarization field overlaid with electrostatic energy mapping with (right) or without (left) vortex domains. Reprinted with permission from Nelson *et al*. [32]. Copyright (2011) American Chemical Society. d) Dark-field TEM image of domain pattern in a BiFeO_3_/SrTiO_3_/BiFeO_3_ tri-layer structure grown on TbScO_3_ substrate, and corresponding schematic of the highlighted region.

Beyond the regular domain structures mentioned above, Nelson *et al.* reported the existence of arrays of spontaneous vortex domains in a 20-nm-thick (001)_P_ BiFeO_3_ thin film on (110)_o_ TbScO_3_ substrate in 2011 [32]. The cross-sectional TEM image and the corresponding schematic in Fig. 2 show the existence of triangle domains at the BiFeO_3_/TbScO_3_ interface where the 109° DWs terminate. Polarization mapping at the triangle domain reveals the formation of a ‘mirrored pair’ of inclined 180° DWs, resulting in a closure vortex structure with polarization rotating around the 109° and 180° DW intersection point. Such a structure is formed due to the existence of bound charge density waves, i.e. alternate positive and negative bound charges at the interfaces, which produce a non-uniform depolarization field (Fig. 2c) favoring the vortex formation. According to our experimental results shown in Fig. 2d, vortex arrays can also be realized at both BiFeO_3_/SrTiO_3_ and the BiFeO_3_/TbScO_3_ interfaces in a BiFeO_3_/SrTiO_3_/BiFeO_3_ heterostructure grown on the (110) TbScO_3_ substrate. Vortex structures were also discovered recently in PdTiO_3_ thin films [60–62] and PdTiO_3_/SrTiO_3_ superlattices [36]. The periodicity of the vortices can be tuned by growing the PbTiO_3_ films with different thicknesses [60]. In ultrathin PbTiO_3_ layers embedded in the PbTiO_3_/SrTiO_3_ superlattices, long-range ordered vortex–antivortex arrays were found to exhibit a nearly continuous polarization rotation [36]. It is interesting to note that as the thickness of the BiFeO_3_ layer decreases, the vortices at the bottom and top interfaces of each BiFeO_3_ layer will move closer to each other. As a result, one can expect to observe an array of new vortices (formed by the merging of the abovementioned vortices located at the bottom and top interfaces) in each BiFeO_3_ layer of the BiFeO_3_/SrTiO_3_ or BiFeO_3_/TbScO_3_ superlattices, similar to the vortex arrays observed in the PbTiO_3_/SrTiO_3_ superlattices.

## DEFECT ENGINEERING

While boundary-condition engineering has demonstrated tremendous success in control of DW patterns in ferroelectric thin films, one major limitation of such a method has been that once the variable at the boundary condition is set, such as the choice of substrate, further modification to control or alter DW patterns during material synthesis becomes difficult. This reduces the parameter space for creating more complex structures with ordered DW patterns and thus imposes restrictions on the functionalities of the system. On the other hand, defects in ferroelectric oxides can also have a remarkable impact on ferroelectric domain structures. Commonly observed defects such as dislocations and vacancies can interact with domains and DWs to pin polarization configurations. The recent work by Li *et al*. [31,40,41] and others [35] reported a strong electrostatic driving force provided by charged impurity defects, another type of common defect with a structure different from the host material. Their results suggested the possibility of using engineered impurity defects in combination with suitable interface boundary conditions to control domain formation and create complex DW structures.

### Planar defects

**Figure 3.**
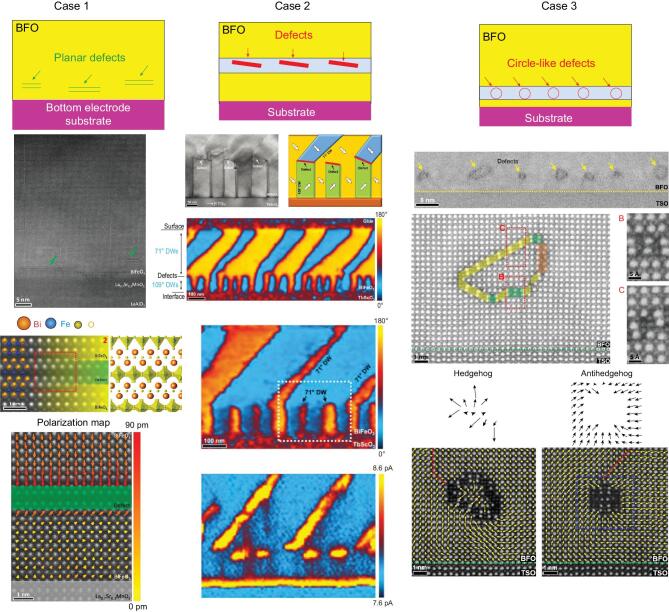
Case 1 shows a planar defect with STEM images and polarization mapping. Reprinted with permission from Li *et al*. [31]. Copyright (2017) American Chemical Society. Case 2 represents circle-like defects with polarization mapping and corresponding defect-region magnified maps. Reprinted with permission from Li *et al*. [40]. Copyright (2017) WILEY-VCH Verlag GmbH & Co. KGaA, Weinheim. Case 3 shows linear defects with TEM and PFM phase images. Reprinted with permission from Li *et al*. [41]. Copyright (2018) American Physical Society.

Li *et al.* initially discovered an array of nanoscale planar charged defect that were accidentally introduced during the synthesis of a BiFeO_3_ film, which can induce a novel mixed-phase ‘head-to-head’ polarization structure [31]. In Fig. 3 case 1, the high-angle annular dark-field (HAADF) image shows brighter and darker dots representing Bi and Fe columns, respectively. Around the defect region Bi_2_FeO_6-x_ formed, consisting of one layer of FeO_6_ octahedra sandwiched by two Bi_2_O_2_ layers. The defect is a few atomic layers thick and is well aligned along the film growth direction. Polarization mapping of the defect region shows head-to-head polarization at the planar defect region. The region above the planar defect has a T-like structure with polarization downward along [00$\bar{1}$]_P_, while the region below has R-like structure with opposing polarization upward along the unit cell diagonal. This upward polarization does not follow the overall downward built-in field that is induced by Schottky contact of BiFeO_3_/La_0.7_Sr_0.3_MnO_3_ [32], indicating the existence of another built-in field pointing upward caused by the defect.

### Linear defects

Inspired by the above finding, Li *et al.* demonstrated a route to deliberately creating an array of linear charged defects in the BiFeO_3_ matrix to configure special DW patterns that are not favorable in natural boundary conditions [40]. By slightly changing the substrate temperature during the film growth, an array of linear charged Fe-rich defects can be introduced into the BiFeO_3_ film at a constant level that is 110–130 nm above the interface (Fig. 3 case 2). The interaction between the charged defects and the polarization lead to a reconfigured DW pattern in the BiFeO_3_ film. As shown in Fig. 3 case 2, a linear defect is observed on each 109° domain that is polarized upward (green) and the horizontal dimension of the defect matches the domain width exactly. Above some of the defects, 71° domains (blue) are stabilized with the DWs located in the ($ 01 \bar{1}$)_P_ planes, which are different from the (101)_P_-oriented 71° domain stripes previously reported in BiFeO_3_ thin films grown on orthorhombic substrates. In contrast, the downward polarized domains (yellow) in the lower portion of the film are not blocked by the defects and can extend their volume to the upper portion. As a result, a transformation from 109° to 71° DW patterns across the defects is observed, as well as an array of ‘head-to-head’ positively charged DWs located exactly at the negatively charged defects. Periodic ordering of these DW patterns is further confirmed by a PFM image of the same heterostructure in the cross section, in which the defects are not resolvable. The PFM image also shows a large 71° domain on top of five 109° domains, with the defects lying in between. The conductivity mapping of the same area was measured and shows the enhanced conductivity of the 71° DWs.

### Circle-like defects

In addition to the planar and linear defects, Li *et al.* found that circle-like defects, named non-stoichiometric nanoregions (NSNRs), can stabilize hedgehog or antihedgehog nanodomains [41]. The circle-like defects were deliberately introduced by changing the substrate temperature during the film growth, similar to the previous example. The stabilized hedgehog/antihedgehog domains can be coupled with polarization rotations, which results in polarization vortices. The hedgehog and antihedgehog domains are shown in Fig. 3 case 3. The formation of the domains is based on non-stoichiometric planar units and stepped units as shown in B and C, respectively, with excess oxygen and result in local negative net charge. The region circled by the defect is a hedgehog domain with a diameter of 4 unit cells in length. The antihedgehog domains with continuous polarization rotation are formed in the region right outside the defect circle and result in flux-closure vortex structure on a large scale.

## DOMAIN WALL CONDUCTIVITY

### 109° and 180° domain walls

**Figure 4.**
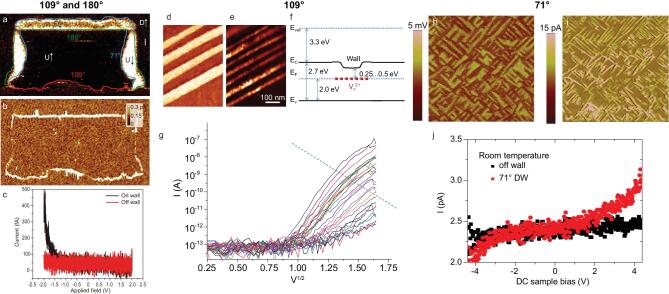
a) In-plane PFM image showing 71° (blue), 109° (red) and 180° (green) DWs. b) Corresponding c-AFM image. c) *I–V* curves taken on the DW (black) and off the DW (red). Reprinted with permission from Seidel *et al*. [15]. Copyright (2009) Springer Nature. d) and e) are PFM phase and c-AFM images of 109° strip domains respectively. f) Schematic band structure of 109° DW. g) *I–V* curves of 109° DW. Reprinted with permission from Seidel *et al*. [16]. Copyright (2010) American Physical Society. h) and i) are PFM phase and c-AFM images of 71° domains. j) *I–V* curves taken on the 71° DW (red) and off the DW (black). Reprinted with permission from Farokhipoor *et al*. [17]. Copyright (2011) American Physical Society.

Seidel *et al*. reported DW conductivity in 2009 [15]. Figure 4a shows the PFM in-plane images of 100-nm-thick BiFeO_3_ thin film on SrRuO_3_ bottom electrode on SrTiO_3_ substrate with a region where 71°, 109° and 180° DWs have been written. The conducting atomic force microscopy (c-AFM) image (Fig. 4b) taken in the same region indicates enhanced electrical conduction at 109° and 180° DWs but not at 71° DWs. The current–voltage (*I*–*V*) curves (Fig. 4c) are measured at the 180° DW or at the middle of the nearby domain region. The black curve reveals that DW has activated conduction while the red curve shows that the domain region has less conduction. They then specifically studied 109° DW conductivity in 2010 [16]. They used 100-nm-thick 10% La-doped BiFeO_3_ thin film, in which La doping allows coherent 109° domain growth [55], with an SrRuO_3_ bottom electrode grown on (110)_o_ DyScO_3_ substrate. PFM and c-AFM results (Fig. 4d and e) indicate an ordered 109° stripe domain structure and corresponding local conductivity at the DWs. The *I*–*V* curves (Fig. 4g) are measured by using AFM tip stepping perpendicular to the 109° DW with the same load. The *I*–*V* curves increase linearly at low voltage and transit to large curvature at higher voltage. Such phenomena were explained by the oxygen vacancy activation in an electric field as shown schematically in Fig. 4f, where enhanced conductivity at the wall is caused by changes in the local bandgap [55].

### 71° domain wall

Although in Seidel *et al*.’s work the conductivity of the 71° DW was not found, Farokhipoor *et al*. reported 71° DW conductivity in 2011 [17]. They studied 40–70-nm-thick BiFeO_3_ thin films with SrRuO_3_ bottom electrode on SrTiO_3_ substrate. The in-plane PFM results (Fig. 4h and i) show that most of the DWs observed are 71° DWs with enhanced conduction. The *I*–*V* curves (Fig. 4j) are measured in constant polarization regions and in regions containing a 71° domain wall. The DW *I*–*V* curve shape (in red in Fig. 4j) reveals a Schottky emission mechanism with the majority of carriers provided by oxygen vacancies. The observed conductivity at 71° DWs here is different from the previous study by Seidel *et al.*, where the 71° DW conductivity is not found in BiFeO_3_ films that were grown on DyScO_3_ substrates at higher deposition rates and with higher oxygen pressure [15]. Such differences indicate that the material synthesis conditions are critical for the conductive nature of different types of DWs.

### Charged domain wall

In ferroelectrics, most DWs are charge-neutral as a result of ‘head-to-tail’ polarization configurations, such as in the previous examples. The electrostatic energy of uncharged DWs is usually minimized but they only have low conductivity that is thermally activated [57]. In contrast, DWs with polarization discontinuity carry net bound charge and are referred to as charged domain walls (CDWs). They are electrically active and can possess much higher conductivity than the charge-neutral DWs. There are two types of CDWs in ferroelectrics. One is formed where the original charge-neutral DW is bent by external applied fields of internal defect pinning effects. Such CDWs have conductivities of ∼ 10–10^3^ times as high as those of bulk domains [7] but tend to reduce to an uncharged state when the applied field is removed or the pinning effects are thermally overcome [63]. The other type is directly formed by ‘head-to-head’ or ‘tail-to-tail’ polarization configuration and can be stabilized by free charge carrier compensation without applied fields or defect pinning [64–66]. These CDWs can have very high conductivity up to 10^9^ times that of the bulk domains and result in an insulating-to-metallic transition [64,67]. Furthermore, the net bound charges can produce a depolarization field that results in an increased electromechanical response and thus better piezoelectric properties [68].

#### Charged domain wall attached to a triangle nanodomain

**Figure 5.**
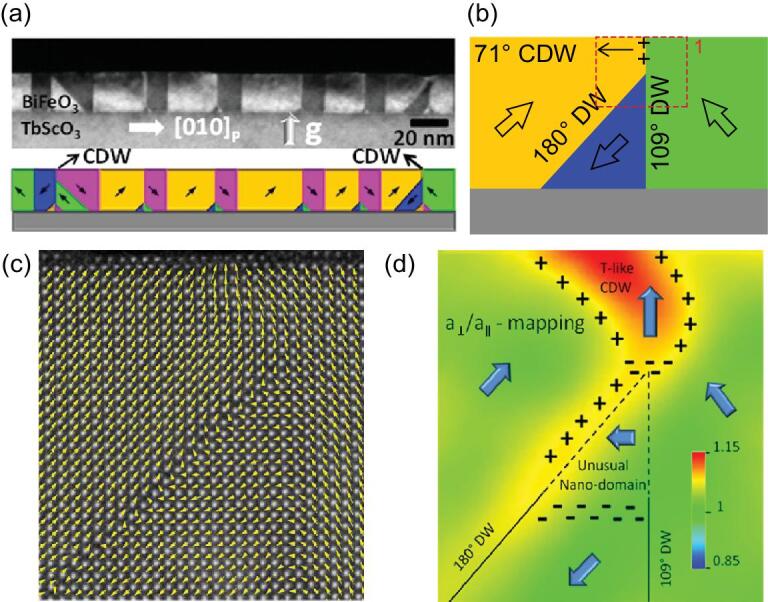
a) Cross-sectional dark-field TEM image showing BiFeO_3_ domain structure. b) CDW region. c) Polarization mapping of a similar area of b). d) Corresponding a$ _{\bot} /{\rm a}_{\parallel} $ mapping. Reprinted with permission from Li *et al*. [56]. Copyright (2013) American Chemical Society.

CDWs attached to triangle domains in BiFeO_3_ film were reported by Li *et al.* [56]. They studied a 20-nm-thick BiFeO_3_ film on a (110)_o_ TbScO_3_ substrate. The triangle domain structure, shown schematically in Fig. 5a, consists of vertical and inclined boundaries that are 109° and 180° DWs, respectively. The 71°CDW above the triangle domain shown in Fig. 5b region 1 is not a linear boundary but an area with gradual polarization rotations (Fig. 5c). Figure 5d shows a large a$ _{\bot} $/a$_{\parallel} $ ratio at the 71°CDW, which indicates the formation of T-like structure at the CDW and a reduction of the electrostatic energy compared to that of a linear ‘head-to-head’ CDW. Also, the T-like 71°CDW region and the surrounding R-like regions result in both positive and compensating negative bound charges, which reduce the electrostatic energy even further.

#### Charged domain wall in a nanoisland

**Figure 6.**
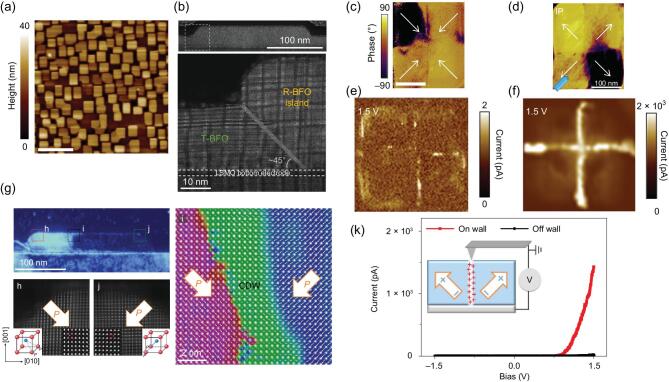
a) Topography of BFO (001) thin film with a self-assembled ordered nanoisland. b) Cross-sectional STEM image of the nanoisland. c) and d) are in-plane PFM images of ‘head-to-head’ and ‘tail-to-tail’ DW structures respectively, e) and f) are corresponding current maps. g) TEM image with enlarged regions h), i), and j). k) *I–V* curve of ‘tail-to-tail’ charged DW. Reprinted with permission from Ma *et al*. [69]. Copyright (2018) Springer Nature.

Ma *et al.* studied CDWs that form in square BiFeO_3_ nanoislands. The film is grown on a 2-nm-thick (La, Sr)MnO_3_ bottom electrode on (001) LaAlO_3_ substrate [69]. As shown in Fig. 6a, their film is composed of a self-assembled, high-density array of square nanoislands. The low-magnification HAADF STEM image (Fig. 6b) shows that the nanoislands are 200 nm in length and 40 nm thick and grow from the 20-nm-thick BiFeO_3_ matrix. High-magnification HAADF STEM images show clear boundaries between the R-like nanoislands and the T-like matrix. The in-plane polarization projection, measured with PFM (Fig. 6c), revealed that each nanoisland has four quadrants that have polarizations all pointing toward the island center. The out-of-plane polarization projection gives polarization pointing downward in the as-grown film. The dark-field TEM image show strong contrast between the left and right regions (Fig. 6g), suggesting different polarization directions. High-magnification HAADF STEM images of regions h, i, and j, highlighted in the low-magnification TEM image, each show different polarization states. Region h has polarization along the body diagonal towards the lower-right center and region j points to the lower-left center. Region i shows a 71° ‘head-to-head’ CDW with 3–5 nm width. Together, these results confirm that the nanoislands contain center-convergent quad-domains formed with 71°CDWs.

As shown in Fig. 6e and f, enhanced conductance with a cross-shape is observed by using c-AFM. Under 1.5 V, the ‘head-to-head’ CDW gives a maximum current of 2 pA. After the quad-domains are switched to form a ‘tail-to-tail’ CDW, the maximum current measured reaches 2 × 10^3^ pA. Such a huge difference between the two types of CDWs results from a difference in their interaction with the bottom electrode's free charge carriers. The free carriers in La_0.7_Sr_0.3_MnO_3_ are p-type, which cannot interact with the positively charged ‘head-to-head’ DW but will accumulate at the negatively charged ‘tail-to-tail’ DW, resulting in very low conductance at ‘head-to-head’ CDWs and enhanced conductance at ‘tail-to-tail’ CDWs. The *I*–*V* curves show diode-type conductance, indicating a large current flow for forward bias, which results from the holes moving from the BiFeO_3_ valence band to the tip. There is no obvious current detected for reverse bias since not enough holes can move to the BiFeO_3_ valence band from the tip.

## DOMAIN WALL DYNAMICS DURING DOMAIN SWITCHING

### Regular thin film

**Figure 7.**
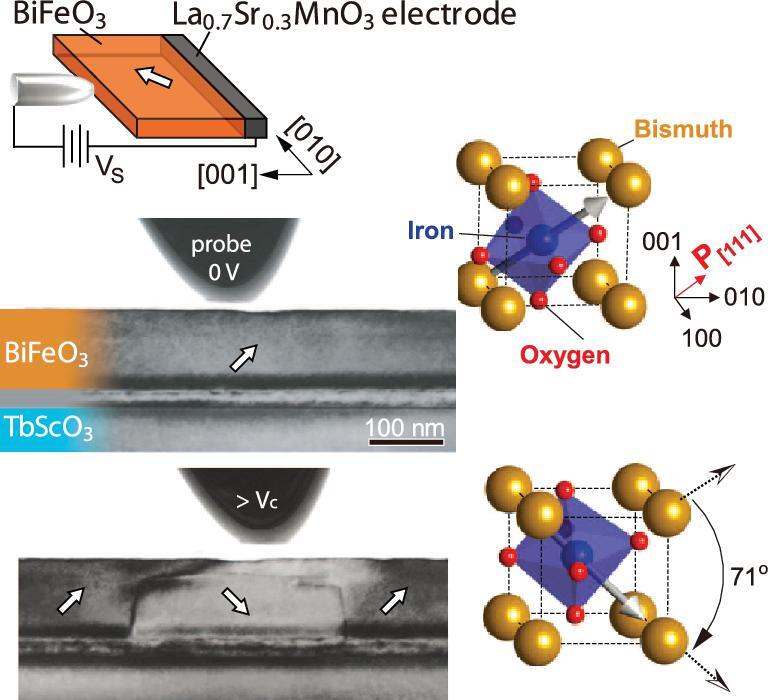
Cross-sectional TEM image of the BiFeO_3_ thin films before and after switching, creating 71° DWs. Reprinted with permission from Nelson *et al.* [70]. Copyright (2011), American Association for the Advancement of Science.

BiFeO_3_ domains can be easily switched by an external electric field, accompanied by DW creation or annihilation. The switching process shown in Fig. 7 was conducted by Nelson *et al.* using a 100 nm-thick (001)_P_ BiFeO_3_ with a 20 nm La_0.7_Sr_0.3_MnO_3_ bottom electrode on TbScO_3_ substrate [70]. The sample starts from a monodomain with polarization along [111]_P_, then a portion of it is switched to a different polarization along [11$\bar{1}$]_P_ by applying a 4 V bias, resulting in the formation of 71° DWs. However, this created domain did not reach the top surface but was pinned midway through the film, forming a horizontal ‘tail-to-tail’ CDW. This written domain can also be erased when applying a reversed bias. These switching processes open a path for properly writing or erasing CDWs.

### Writing and erasing charged domain walls

**Figure 8.**
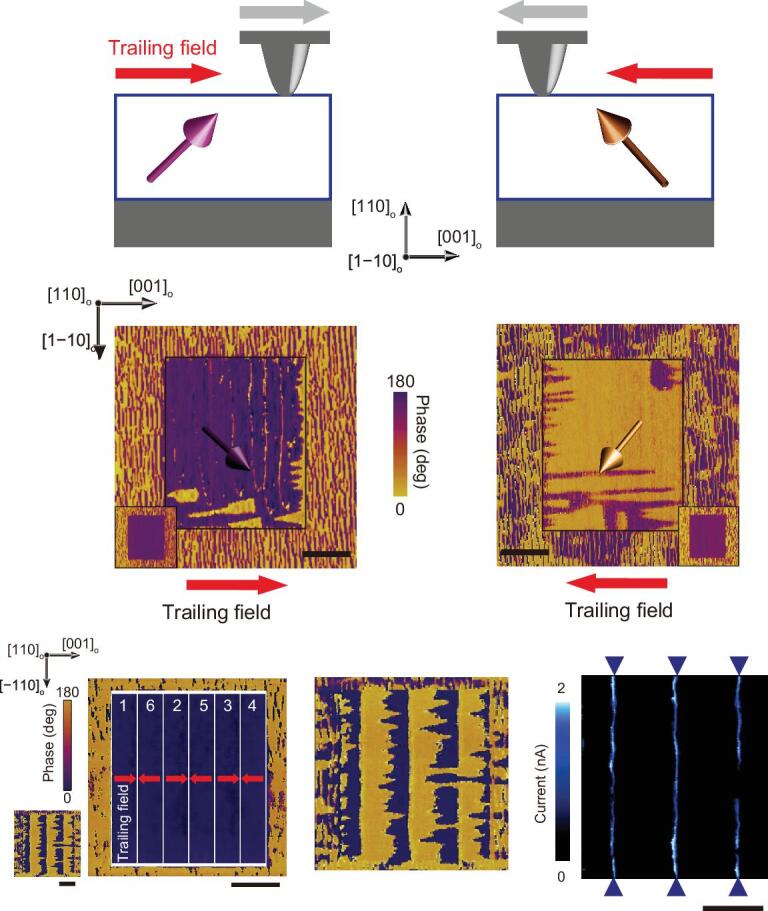
In-plane PFM phase and current mapping of BiFeO_3_ thin films showing the writing of ‘head-to-head’ and ‘tail-to-tail’ charged DWs. Reprinted with permission from Crassous *et al*. [9]. Copyright (2015) Springer Nature.

With the understanding of the CDW formation and fundamental mechanisms, Crassous *et al.* reported a technique to easily write and erase CDWs in 45-nm-thick La-doped BiFeO_3_ thin films with an SrRuO_3_ bottom electrode on (110)_o_ DyScO_3_ substrate using PFM [9]. As the tip is moving along the BiFeO_3_ surface, it creates a trailing field [71] that controls the in-plane polarization direction while the perpendicular electric field between the tip and bottom electrode controls the out-of-plane polarization direction, as shown in Fig. 8. Two 3 × 3 μm^2^ regions were initially written into a monodomain with different trailing fields to demonstrate CDW writing. The writing process is initiated by applying an electric field to pull the out-of-plane polarization up combined with the trailing field to control the in-plane polarization direction. Domains 1, 2 and 3 are written first with the trailing field oriented along the [001]_o_ direction and then domains 4, 5 and 6 are written with the trailing field along the opposite direction, so that domains 1–3 are polarized right and domains 4–6 are polarized left. The in-plane PFM results show ‘head-to-head’ CDWs at the 1/6, 2/5 and 3/4 interfaces, and ‘tail-to-tail’ CDWs at the 6/2 and 5/3 interfaces. The c-AFM mapping shows a current of ∼ 1.7 nA at the ‘head-to-head’ CDW with a 2.5 V bias applied. No conductance is detected at the ‘tail-to-tail’ CDW. Interestingly, such observations are totally different from the nanoisland CDW results reported by Ma *et al.* Crassous *et al.* believe that this is because the positive bound charges at the ‘head-to-head’ CDW are screened by the electrons from the tip and result in the creation of a conductive state. The ‘tail-to-tail’ CDW, on the other hand, has negative bound charges at the wall that cannot be compensated by the free electrons from the tip and therefore tend to minimize its charged density by forming zigzag DWs. Also, in Crassous *et al.*’s case, SrRuO_3_ bottom electrodes (*n*-type) are used, providing mobile electrons to screen the positive bound charges at the ‘head-to-head’ CDWs, while Ma *et al.* used (La, Sr)MnO_3_, which provides mobile holes, resulting in conducting ‘tail-to-tail’ CDWs as mentioned in section ‘Charged domain wall in a nanoisland’.

**Figure 9.**
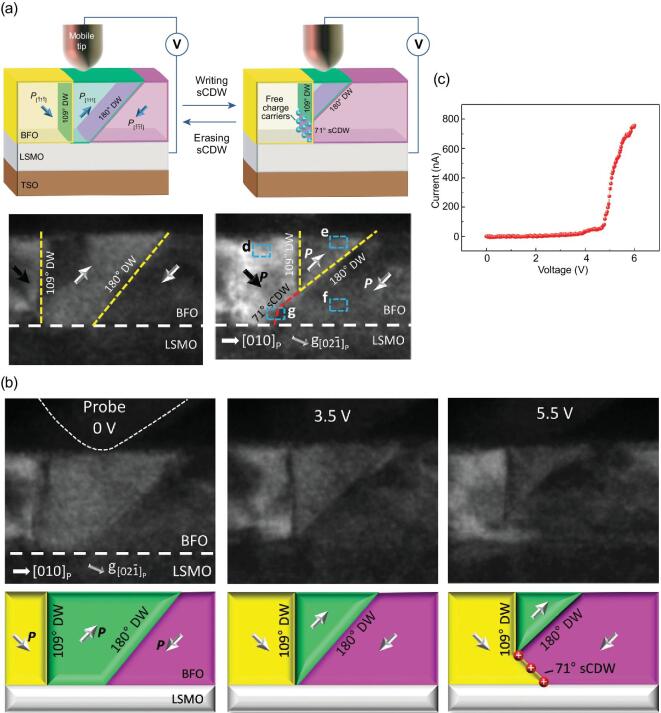
a) Schematic of the charged DW writing and erasing process with corresponding TEM images. b) *In-situ* switching of CDW writing. c) Corresponding *I–V* curve. Reprinted with permission from Li *et al*. [57]. Copyright (2016) WILEY-VCH Verlag GmbH & Co. KGaA, Weinheim.

The manipulation of CDWs through scanning probe microscopy (such as PFM) only allows access to the domain structure from the surface. Due to the requirement of free charge compensation, however, some CDWs present at the top surface of ferroelectric films may not penetrate to the bottom interface. With cross-sectional *in situ* TEM, Li *et al*. reported a detailed study of local CDW writing and erasing in a 20-nm-thick (001)_P_ BiFeO_3_ thin film with an La_0.7_Sr_0.3_MnO_3_ bottom electrode on (110)_o_ TbScO_3_ substrates [57]. The experimental set-up and writing process are schematically shown in Fig. 9a, with vertical 109° and inclined 180° DWs, and the corresponding cross-sectional dark-field TEM images are shown in the bottom row. In Fig. 9a, the left configuration is stable without CDW because the 109° and 180° DWs are far away from each other and therefore have very weak electrostatic interaction. Once the CDW has been written, the system is still stable since the bound charge close to the interface can be compensated by free carriers from the bottom electrode, reducing the electrostatic energy. The process was induced by applying an external electric field. The 109° and 180° DWs start to move as the bias reaches 1.7 V. While the bias is increasing, the two domains move toward each other and intersect, which eventually results in a shrinkage of the triangular domain and the formation of a CDW. The corresponding *I*–*V* curve in Fig. 9c reveals that the film has no conductivity below 3.5 V when there is no CDW, but passes 640 nA at 5.5 V once the CDW has formed. Such results give rise to the correlation between the enhanced conductance and the existence of CDW.

## DOMAIN WALL PHOTOVOLTAICS

**Figure 10.**
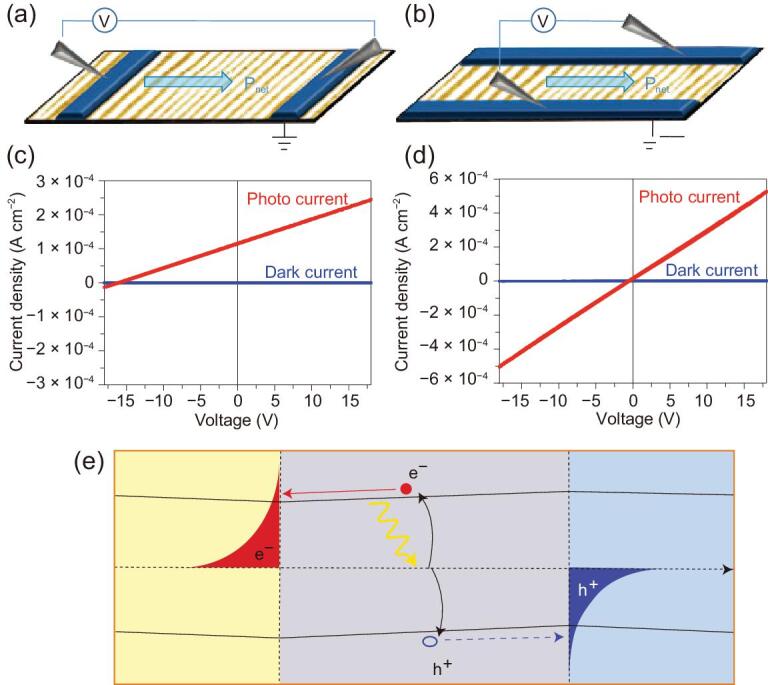
Geometries with electrodes (a) parallel and (b) perpendicular to the DWs and corresponding *I–V* curves (c) and (d). (e) The schematic of the charge separation model. Reprinted with permission from Yang *et al*. [20]. Copyright (2010) Springer Nature.

While the conventional light-to-electrical energy conversion process is achieved by using a *p*–*n* junction or a heterojunction [72,73] to separate electrons and holes, Yang *et al.* developed a new model for charge separation and photovoltage generation using BiFeO_3_ ferroelectric DW modification under white light, yielding photovoltage significantly higher than the bandgap [20]. BiFeO_3_ has a direct bandgap of ∼ 2.67 eV [74] with open-circuit voltage *V*_oc_$\ll$*E*_g_. The photovoltaic nanodevices are fabricated by defining two 500-μm-long platinum electrodes separated by 200 μm, on top of a100-nm-thick BiFeO_3_ film; the electrodes can be either parallel or perpendicular to the DWs (Fig. 10a, b). Figure 10c, d shows the measured *I*–*V* curves of 71° DWs with both electrode configurations under no illumination and saturation illumination with white light. In the parallel configuration, *V*_oc_ is measured as 16 V with an in-plane short-circuit current density of ∼ 1.2 × 10^−4^ A cm^−2^. The perpendicular configuration, on the other hand, shows large photoconductivity but no *V*_oc_. A drop in potential was measured across all types of DWs, with 180° DWs showing the largest drop, followed by 109° and then 71° DWs. They believe that such phenomena can be explained by the schematic band structure model in Fig. 10e depicting both the domains and DWs in dark conditions. The drop in potential across the DW creates an electric field that will provide an enhanced electron–hole pair separation process when illuminated. The separated electrons and holes build up on either side of the DWs and sum up over a large scale, leading to the large *V*_oc_. In contrast, if the light is incident inside the domain, the excited charge is localized and tightly bound due to the weak built-in electrical field, resulting in a quick recombination and no photovoltaic effect.

**Figure 11.**
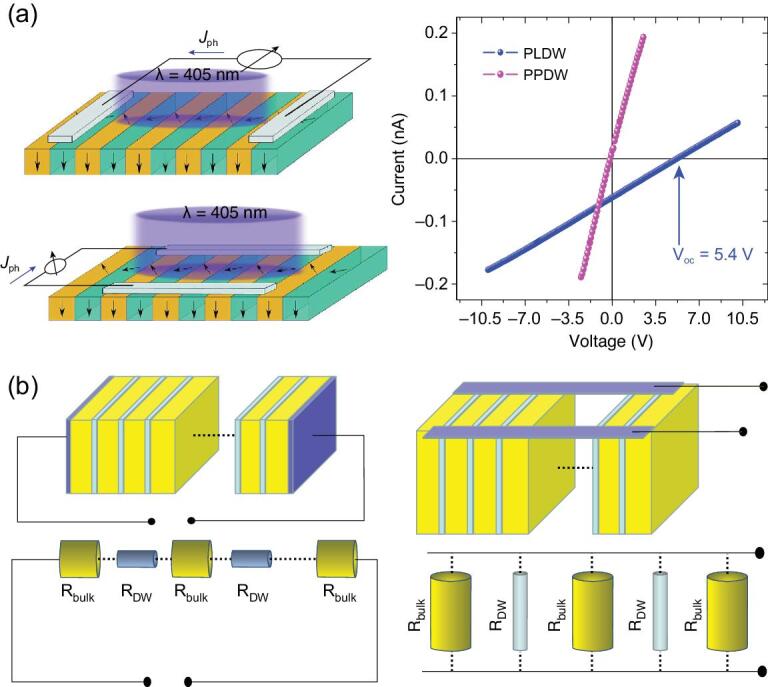
a) Geometries with electrodes parallel and perpendicular to the DWs and corresponding *I–V* curves: electrodes parallel to the DWs (PLDW) and perpendicular to the DWs (PPDW). b) A schematic of the role of the DWs. Reprinted with permission from Bhatnagar *et al*. [22]. Copyright (2013) Springer Nature.

This model, however, has been challenged by Bhatnagar *et al.*’s work with a measurement combing photoelectric AFM and PFM at the DWs and inside domains on 100 nm-thick (001)_P_ BiFeO_3_ thin films grown on (110)_o_ TbScO_3_ substrate [22]. They studied the BiFeO_3_ photovoltaic effect on both 71° and 109° DWs, and the experimental set up is similar to the schematics in Fig. 11a with Pt electrodes that are 250 μm in length. The *I*–*V* curves of 109° DWs with parallel geometry give *V*_oc_ of 5.4 V, which is larger than the BiFeO_3_ bandgap. For 71° DWs, the *I*–*V* curves of both parallel and perpendicular geometries show significant *V*_oc_ of −7.6 V and − 6.6 V, respectively. Both groups’ results show a *V*_oc_ larger than the BiFeO_3_ bandgap, but Bhatnagar *et al.*’s work claims that the DWs, due to their relatively high intrinsic conductivity, work as resistors with lower resistance rather than working to separate charges as mentioned in Yang *et al*.’s model. The relationship between *V*_oc_ and the conductivity can be expressed by the following equation:

[75–77] $$\begin{equation*}
{V_{{\rm{oc}}}} = {J_{{\rm{ph}}}}\ \left( {\frac{1}{{{\sigma _{\rm{d}}} + {\sigma _{{\rm{ph}}}}}}} \right)L,
\end{equation*}$$

where *J*_ph_ is the photocurrent, σ_d_ is the dark conductivity, σ_ph_ is the photoconductivity, and *L* is the distance between the electrodes. Only the dark conductivity and the photoconductivity affect *V*_oc_, since *J*_ph_ is uniformly generated across the whole film by shift currents [78], and *L* is constant. In Fig. 11b, the total effective conductivity (${\sigma _{\rm{d}}} + {\sigma _{{\rm{ph}}}}$) has contributions from both the domains and the DWs, which can be modeled as either a parallel or serial circuit. The perpendicular geometry gives lower *V*_oc_ since it has parallel conductivity mode and results in higher conductivity. Also, the 109° DW has higher conductivity than that of the 71° DW, which results in smaller *V*_oc_ for the 109° DW than that of 71° DWs, in all cases.

In aggrement with Bhatnagar *et al.*’s model, Wang *et al.* reported the photovoltic effect on BiFeO_3_ CDWs [23], which is a follow-up work to section ‘Charged domain wall in a nanoisland’. They used a similar square BiFeO_3_ nanoisland with either ‘head-to-head’ or ‘tail-to-tail’ CDWs configurations as shown in Fig. 6c and d, repectively, and photoassisted AFM to measure the photoresponse behavior [79]. As the 1.4 V bias voltage is applied, the nanoisland with ‘head-to-head’ CDWs shows ∼ 0.2 pA dark current, and 23 pA photocurrent when irradiated with a 405 nm laser, giving a photo-to-dark current on–off ratio of 11,500%. In contrast, ‘tail-to-tail’ CDWs show 0.69 nA dark current and 1.1 nA photocurrent, with a much lower on–off ratio of 159%. The measurements at the domains without/with laser illumination show negligible signal.

## SUMMARY AND PROSPECTS

Here, we have reviewed the current status of BiFeO_3_ DW microstructures and their conductive and photovoltaic properties. The uncharged DWs show enhanced conductance with measured current in the pA range, while the later reported CDWs have conductances of four orders of magnitude higher than the uncharged DWs. The ferroelectric switching process in CDWs opens up a way to directly manipulate their formation and motion, forming the building blocks for reconfigurable nanoelectronic devices based on writing and erasing CDWs. The role of DWs in producing photovoltaic properties is important: running parallel to the collecting electrodes provides a high open-circuit voltage larger than the BiFeO_3_ bandgap. Many other DW properties and mechanisms are, however, still unclear and need to be explored, such as stress–polarization bound charge coupling, electron–hole pair dynamics, defect engineering with larger impurity defect dimensions, and switching processes with higher spatial resolution. To achieve this, additional characterization with TEM works is necessary.

Because of the high spatial resolution and flexibility for housing *in situ* probes, TEM has been used for studies on the atomic structure, dynamics and electronic structure of ferroelectric materials, especially heterogeneous structures such as interfaces and DWs. While unsettled mechanisms, such as point defect and oxygen vacancy dynamics, represent new challenges for characterization, the emergence of new imaging techniques may bring new possibilities to these studies. The advanced 4D STEM technique including charge density mapping may provide atomic-level local charge and electric field distribution at the DWs, giving rise to better understanding of bound charge coupling and electron–hole pair dynamics. The cross-sectional c-AFM imaging technique [40,80] plus cross-sectional STEM provides both macro- and micro-level information that can be applied to large defect dimension studies. Vibrational microscopy and high-energy-resolution EELS, which can detect oxygen vacancies and electronic structures on the atomic scale, may help to provide more information on and better explanations for many current problems. For DW switching, only low-magnification TEM images have been available thus far because most *in situ* sample holders are limited to a single tilt axis, making it impossible to accurately align the microscope onto the crystal's zone axis, a requirement for atomic resolution imaging. The new double tilt *in situ* TEM holder developed by Mingjie Xu *et al.* [81] opens up a possible path to tilt the sample to the zone axis, enabling the acquisition of high-magnification atomic-scale switching images. This may help improve our understanding of DW formation and the interaction with surrounding defects during the process.

## References

[1] Vonhippel A , Breckenridge RG, Chesley FG et al. High dielectric constant ceramics. Ind Eng Chem 1946; 38: 1097–109.

[2] Sergei VK , Anna NM, Long Qing C et al. Local polarization dynamics in ferroelectric materials. Rep Prog Phys 2010, 73: 056502.

[3] Ishiwara H . Ferroelectric random access memories. J Nanosci Nanotechnol 2012; 12: 7619–27. 23421123 10.1166/jnn.2012.6651

[4] Streiffer SK , Parker CB, Romanov AE et al. Domain patterns in epitaxial rhombohedral ferroelectric films. I. Geometry and experiments. J Appl Phys 1998; 83: 2742–53.

[5] Chu YH , Zhan Q, Martin LW et al. Nanoscale domain control in multiferroic BiFeO_3_ thin films. Adv Mater 2006; 18: 2307–11.

[6] De Luca G , Strkalj N, Manz S et al. Nanoscale design of polarization in ultrathin ferroelectric heterostructures. Nat Commun 2017; 8: 1419. 29127282 10.1038/s41467-017-01620-2 PMC5681682

[7] Maksymovych P , Morozovska AN, Yu P et al. Tunable metallic conductance in ferroelectric nanodomains. Nano Lett 2012; 12: 209–13. 22181709 10.1021/nl203349b

[8] Vasudevan RK , Morozovska AN, Eliseev EA et al. Domain wall geometry controls conduction in ferroelectrics. Nano Lett 2012; 12: 5524–31. 22994244 10.1021/nl302382k

[9] Crassous A , Sluka T, Tagantsev AK et al. Polarization charge as a reconfigurable quasi-dopant in ferroelectric thin films. Nat Nanotechnol 2015; 10: 614–8. 26076468 10.1038/nnano.2015.114

[10] Solmaz A , Huijben M, Koster G et al. Domain selectivity in BiFeO_3_ thin films by modified substrate termination. Adv Funct Mater 2016; 26: 2882–9.

[11] Paruch P , Giamarchi T, Triscone JM. Domain wall roughness in epitaxial ferroelectric PbZr_0.2_Ti_0.8_O_3_ thin films. Phys Rev Lett 2005; 94: 197601. 16090210 10.1103/PhysRevLett.94.197601

[12] Lubk A , Rossell MD, Seidel J et al. Evidence of sharp and diffuse domain walls in BiFeO_3_ by means of unit-cell-wise strain and polarization maps obtained with high resolution scanning transmission electron microscopy. Phys Rev Lett 2012; 109: 047601. 23006107 10.1103/PhysRevLett.109.047601

[13] Vrejoiu I , Le Rhun G, Zakharov ND et al. Threading dislocations in epitaxial ferroelectric PbZr_0.2_Ti_0.8_O_3_ films and their effect on polarization backswitching. Philos Mag 2006; 86: 4477–86.

[14] Rojac T , Bencan A, Drazic G et al. Domain-wall conduction in ferroelectric BiFeO_3_ controlled by accumulation of charged defects. Nat Mater 2017; 16: 322–7. 27842075 10.1038/nmat4799

[15] Seidel J , Martin LW, He Q et al. Conduction at domain walls in oxide multiferroics. Nat Mater 2009; 8: 229–34. 19169247 10.1038/nmat2373

[16] Seidel J , Maksymovych P, Batra Y et al. Domain wall conductivity in La-doped BiFeO_3_. Phys Rev Lett 2010; 105: 197603. 21231197 10.1103/PhysRevLett.105.197603

[17] Farokhipoor S , Noheda B. Conduction through 71° domain walls in BiFeO_3_ thin films. Phys Rev Lett 2011; 107: 127601. 22026801 10.1103/PhysRevLett.107.127601

[18] Sharma P , Zhang Q, Sando D et al. Nonvolatile ferroelectric domain wall memory. Sci Adv 2017; 3: e1700512. 28691100 10.1126/sciadv.1700512 PMC5482552

[19] Jiang J , Bai ZL, Chen ZH et al. Temporary formation of highly conducting domain walls for non-destructive read-out of ferroelectric domain-wall resistance switching memories. Nat Mater 2018; 17: 49–56. 29180776 10.1038/nmat5028

[20] Yang SY , Seidel J, Byrnes SJ et al. Above-bandgap voltages from ferroelectric photovoltaic devices. Nat Nanotechnol 2010; 5: 143–7. 20062051 10.1038/nnano.2009.451

[21] Alexe M , Hesse D. Tip-enhanced photovoltaic effects in bismuth ferrite. Nat Commun 2011; 2: 256.

[22] Bhatnagar A , Roy Chaudhuri A, Heon Kim Y et al. Role of domain walls in the abnormal photovoltaic effect in BiFeO_3_. Nat Commun 2013; 4: 2835.

[23] Wang J , Ma J, Yang Y et al. Ferroelectric photodetector with high current on–off ratio (∼1 × 104%) in self-assembled topological nanoislands. ACS Appl Electron Mater 2019; 1: 862–8.

[24] Wang F , Young SM, Zheng F et al. Substantial bulk photovoltaic effect enhancement via nanolayering. Nat Commun 2016; 7: 10419. 26791545 10.1038/ncomms10419 PMC4735945

[25] Inoue R , Ishikawa S, Imura R et al. Giant photovoltaic effect of ferroelectric domain walls in perovskite single crystals. Sci Rep 2015; 5: 14741. 26443381 10.1038/srep14741 PMC4595799

[26] Chu K , Jang B-K, Sung JH et al. Enhancement of the anisotropic photocurrent in ferroelectric oxides by strain gradients. Nat Nanotechnol 2015; 10: 972–9. 26322941 10.1038/nnano.2015.191

[27] Schaab J , Skjærvø SH, Krohns S et al. Electrical half-wave rectification at ferroelectric domain walls. Nat Nanotechnol 2018; 13: 1028–34. 30201990 10.1038/s41565-018-0253-5

[28] Zavaliche F , Yang SY, Zhao T et al. Multiferroic BiFeO_3_ films: domain structure and polarization dynamics. Phase Transitions 2006; 79: 991–1017.

[29] Zeches RJ , Rossell MD, Zhang JX et al. A strain-driven morphotropic phase boundary in BiFeO_3_. Science 2009; 326: 977–80. 19965507 10.1126/science.1177046

[30] Christen HM , Nam JH, Kim HS et al. Stress-induced R-MA-MC-T symmetry changes in BiFeO_3_ films. Phys Rev B 2011; 83: 144107.

[31] Li LZ , Zhang Y, Xie L et al. Atomic-scale mechanisms of defect-induced retention failure in ferroelectrics. Nano Lett 2017; 17: 3556–62. 28471679 10.1021/acs.nanolett.7b00696

[32] Nelson CT , Winchester B, Zhang Y et al. Spontaneous vortex nanodomain arrays at ferroelectric heterointerfaces. Nano Lett 2011; 11: 828–34. 21247184 10.1021/nl1041808

[33] Yun KY , Ricinschi D, Kanashima T et al. Giant ferroelectric polarization beyond 150 μC/cm^2^ in BiFeO_3_ thin film. Jpn J Appl Phys 2004; 43: L647–8.

[34] Wang J , Neaton JB, Zheng H et al. Epitaxial BiFeO_3_ multiferroic thin film heterostructures. Science 2003; 299: 1719–22. 12637741 10.1126/science.1080615

[35] Vasudevan RK , Chen Y-C, Tai H-H et al. Exploring topological defects in epitaxial BiFeO_3_ thin films. ACS Nano 2011; 5: 879–87. 21214267 10.1021/nn102099z

[36] Yadav AK , Nelson CT, Hsu SL et al. Observation of polar vortices in oxide superlattices. Nature 2016; 530: 198–201. 26814971 10.1038/nature16463

[37] Schlom DG , Chen L-Q, Eom C-B et al. Strain tuning of ferroelectric thin films. Annu Rev Mater Res 2007; 37: 589–626.

[38] Schlom DG , Chen L-Q, Pan X et al. A thin film approach to engineering functionality into oxides. J Am Ceram Soc 2008; 91: 2429–54.

[39] Martin LW , Chu YH, Ramesh R. Advances in the growth and characterization of magnetic, ferroelectric, and multiferroic oxide thin films. Mater Sci Eng R Rep 2010; 68: 89–133.

[40] Li L , Jokisaari JR, Zhang Y et al. Control of domain structures in multiferroic thin films through defect engineering. Adv Mater 2018; 30: 1802737. 10.1002/adma.201802737 30084144

[41] Li L , Cheng X, Jokisaari JR et al. Defect-induced hedgehog polarization states in multiferroics. Phys Rev Lett 2018; 120: 137602. 29694202 10.1103/PhysRevLett.120.137602

[42] Chen Z , Liu J, Qi Y et al. 180° ferroelectric stripe nanodomains in BiFeO_3_ thin films. Nano Lett 2015; 15: 6506–13. 26317408 10.1021/acs.nanolett.5b02031

[43] Choi KJ , Biegalski M, Li YL et al. Enhancement of ferroelectricity in strained BaTiO_3_ thin films. Science 2004; 306: 1005–9. 15528439 10.1126/science.1103218

[44] Ederer C , Spaldin NA. Effect of epitaxial strain on the spontaneous polarization of thin film ferroelectrics. Phys Rev Lett 2005; 95: 257601. 16384507 10.1103/PhysRevLett.95.257601

[45] Schlom D , Gv Chen L-Q, Fennie CJ et al. Elastic strain engineering of ferroic oxides. MRS Bull 2014; 39: 118–30.

[46] Adamo C , Ke X, Wang HQ et al. Effect of biaxial strain on the electrical and magnetic properties of (001) La_0.7_Sr_0.3_MnO_3_ thin films. Appl Phys Lett 2009; 95: 112504.

[47] Infante IC , Lisenkov S, Dupe B et al. Bridging multiferroic phase transitions by epitaxial strain in BiFeO_3_. Phys Rev Lett 2010; 105: 057601. 20867953 10.1103/PhysRevLett.105.057601

[48] Bea H , Dupe B, Fusil S et al. Evidence for room-temperature multiferroicity in a compound with a giant axial ratio. Phys Rev Lett 2009; 102: 217603. 19519136 10.1103/PhysRevLett.102.217603

[49] Chisholm MF , Luo WD, Oxley MP et al. Atomic-scale compensation phenomena at polar interfaces. Phys Rev Lett 2010; 105: 197602. 21231196 10.1103/PhysRevLett.105.197602

[50] Lichtensteiger C , Fernandez-Pena S, Weymann C et al. Tuning of the depolarization field and nanodomain structure in ferroelectric thin films. Nano Lett 2014; 14: 4205–11. 24983128 10.1021/nl404734z

[51] Li YL , Hu SY, Liu ZK et al. Effect of electrical boundary conditions on ferroelectric domain structures in thin films. Appl Phys Lett 2002; 81: 427–9.

[52] Chu YH , Cruz MP, Yang CH et al. Domain control in multiferroic BiFeO_3_ through substrate vicinality. Adv Mater 2007; 19: 2662–6.

[53] Jang HW , Ortiz D, Baek SH et al. Domain engineering for enhanced ferroelectric properties of epitaxial (001) BiFeO_3_ thin films. Adv Mater 2009; 21: 817–23.

[54] Folkman CM , Baek SH, Jang HW et al. Stripe domain structure in epitaxial (001) BiFeO_3_ thin films on orthorhombic TbScO_3_ substrate. Appl Phys Lett 2009; 94: 251911.

[55] Chu Y-H , He Q, Yang C-H et al. Nanoscale control of domain architectures in BiFeO_3_ thin films. Nano Lett 2009; 9: 1726–30. 19351199 10.1021/nl900723j

[56] Li LZ , Gao P, Nelson CT et al. Atomic scale structure changes induced by charged domain walls in ferroelectric materials. Nano Lett 2013; 13: 5218–23. 24070735 10.1021/nl402651r

[57] Li LZ , Britson J, Jokisaari JR et al. Giant resistive switching via control of ferroelectric charged domain walls. Adv Mater 2016; 28: 6574–80. 27213756 10.1002/adma.201600160

[58] Nesterov O , Matzen S, Magen C et al. Thickness scaling of ferroelastic domains in PbTiO_3_ films on DyScO_3_. Appl Phys Lett 2013; 103: 142901.

[59] Catalan G , Janssens A, Rispens G et al. Polar domains in lead titanate films under tensile strain. Phys Rev Lett 2006; 96: 127602. 16605960 10.1103/PhysRevLett.96.127602

[60] Tang YL , Zhu YL, Ma XL et al. Observation of a periodic array of flux-closure quadrants in strained ferroelectric PbTiO_3_ films. Science 2015; 348: 547–51. 25883317 10.1126/science.1259869

[61] Liu Y , Wang Y-J, Zhu Y-L et al. Large scale two-dimensional flux-closure domain arrays in oxide multilayers and their controlled growth. Nano Lett 2017; 17: 7258–66. 29125773 10.1021/acs.nanolett.7b02615

[62] Li S , Wang YJ, Zhu YL et al. Evolution of flux-closure domain arrays in oxide multilayers with misfit strain. Acta Mater 2019; 171: 176–83.

[63] Gao P , Nelson CT, Jokisaari JR et al. Direct observations of retention failure in ferroelectric memories. Adv Mater 2012; 24: 1106–10. 22331626 10.1002/adma.201103983

[64] Sluka T , Tagantsev AK, Bednyakov P et al. Free-electron gas at charged domain walls in insulating BaTiO_3_. Nat Commun 2013; 4: 1808. 23651996 10.1038/ncomms2839 PMC3674246

[65] Vul BM , Guro GM, Ivanchik II. Encountering domains in ferroelectrics. Ferroelectrics 1973; 6: 29–31.

[66] Gureev MY , Tagantsev AK, Setter N. Head-to-head and tail-to-tail 180° domain walls in an isolated ferroelectric. Phys Rev B 2011; 83: 184104.

[67] Bednyakov PS , Sluka T, Tagantsev AK et al. Formation of charged ferroelectric domain walls with controlled periodicity. Sci Rep 2015; 5: 15819. 26516026 10.1038/srep15819 PMC4626787

[68] Li L , Britson J, Jokisaari JR et al. Giant resistive switching via control of ferroelectric charged domain walls. Adv Mater 2016; 28: 6574–80. 27213756 10.1002/adma.201600160

[69] Ma J , Ma J, Zhang Q et al. Controllable conductive readout in self-assembled, topologically confined ferroelectric domain walls. Nat Nanotechnol 2018; 13: 947–52. 30038370 10.1038/s41565-018-0204-1

[70] Nelson CT , Gao P, Jokisaari JR et al. Domain dynamics during ferroelectric switching. Science 2011; 334: 968–71. 22096196 10.1126/science.1206980

[71] Balke N , Choudhury S, Jesse S et al. Deterministic control of ferroelastic switching in multiferroic materials. Nat Nanotechnol 2009; 4: 868–75. 19893529 10.1038/nnano.2009.293

[72] Gur I , Fromer NA, Geier ML et al. Air-stable all-inorganic nanocrystal solar cells processed from solution. Science 2005; 310: 462–5. 16239470 10.1126/science.1117908

[73] O’Regan B , Grätzel M. A low-cost, high-efficiency solar cell based on dye-sensitized colloidal TiO_2_ films. Nature 1991; 353: 737–40.

[74] Basu SR , Martin LW, Chu YH et al. Photoconductivity in BiFeO_3_ thin films. Appl Phys Lett 2008; 92: 091905.

[75] Chynoweth AG . Surface space-charge layers in barium titanate. Phys Rev 1956; 102: 705–14.

[76] Ruppel W , Von Baltz R, Wurfel P. The origin of the photo-emf in ferroelectric and non-ferroelectric materials. Ferroelectrics 1982; 43: 109–23.

[77] Fridkin VM . Photoferroelectrics. Berlin Heidelberg: Springer-Verlag, 1979.

[78] Young SM , Zheng F, Rappe AM. First-principles calculation of the bulk photovoltaic effect in bismuth ferrite. Phys Rev Lett 2012; 109: 236601. 23368233 10.1103/PhysRevLett.109.236601

[79] Wang J , Huang H, He W et al. Nanoscale bandgap tuning across an inhomogeneous ferroelectric interface. ACS Appl Mater Interfaces 2017; 9: 24704–10. 28686410 10.1021/acsami.7b05138

[80] Zhang Y , Lu H, Xie L et al. Anisotropic polarization-induced conductance at a ferroelectric–insulator interface. Nat Nanotechnol 2018; 13: 1132–6. 30250247 10.1038/s41565-018-0259-z

[81] Xu M , Dai S, Blum T et al. Double-tilt in situ TEM holder with ultra-high stability. Ultramicroscopy 2018; 192: 1–6. 29800933 10.1016/j.ultramic.2018.04.010

